# Cross‐Sectional Study on Experience in Musical Instruments and Basic Surgical Skills Acquisition Among Final Year Medical Students

**DOI:** 10.1111/ans.70698

**Published:** 2026-04-23

**Authors:** Michael Tiong Hong Co, Billy Ho Hung Cheung, Katrina Kwan Ching Chan

**Affiliations:** ^1^ Centre for Education and Learning, Department of Surgery University of Hong Kong Hong Kong SAR China

## Abstract

**Introduction:**

This cross‐sectional study aims to evaluate the association between experience in musical instruments and surgical skills in medical students.

**Methods:**

Online questionnaires were distributed to 243 final year students for their baseline demographic data and exposure to musical instrument training. A two‐hour surgical skills training was conducted, and students were assessed for their surgical skills by two blinded assessors 4 weeks after the teaching using the objective structured assessment of technical skills (OSATS) score.

**Results:**

Two hundred three final year medical students were recruited. The median age was 24 years old (range 23–34). 126 (62.3%) had experience in playing musical instruments. 101 (80.2%) students mainly played keyboard, 23 (18.3%) played strings, and 2 (1.5%) played brass/wind. The mean time for task completion was 249.3 s (SD = 45.9) in the control group (no musical experience), while the case group (with musical experience) was 223.5 s (SD = 28.8), *p* < 0.001. The mean OSATS score in the control group was 7.79 (SD = 0.78) while the case group was 8.28 (SD = 0.59), *p* < 0.001. The mean time for task completion was 223.8 s (SD = 30.8) in the keyboard group, while that in the strings group was 223.2 s (SD = 18.6), *p* = 0.926. The mean OSATS score was 8.3 (SD = 0.59) in the keyboard group and 8.26 in the strings group (SD = 0.54), *p* = 0.789.

**Conclusion:**

Experience with musical instruments is associated with quicker time of basic surgical tasks completion with better technique.

## 1 Introduction

The acquisition of surgical skills requires extensive psychomotor and visuospatial skills [1, 2]. This reflects the tremendous amount of time required for surgeons in training to reach a satisfactory amount of technical competency. With the increasing demand for surgical services owing to the growing aging population and the ever‐rising expectations of surgical procedures, various research projects were published, trying to identify predictors or factors affecting surgical competency, in hope of searching for potential medical graduates with intrinsic abilities to achieve good surgical skills. Amongst these studies, musical instrument playing is one of the most studied potential predictors, as it also requires complex visuospatial skills similar to those required in surgical practice [3]. Existing studies have controversial findings regarding the impact of musical instrument playing and the acquisition of surgical skills, the majority of which either show a positive association or no association.

Many studies have found a positive association between musical instrument playing and surgical skills acquisition. A cross‐sectional study by Sun et al., which looked at the influence of musical background on surgical skills acquisition amongst undergraduates and medical trainees without prior exposure to surgical training, found that musical background was associated with better fundamental surgical skills performance [1]. Musicality was assessed by a survey and a Mini‐Profile of Music Perception Skills test, and participants with prior musical instrument playing exposure were grouped into playing either the Piano, Guitar, Wind, or Strings [1]. Surgical skills performance was mainly reflected by asking participants to complete several tasks which reflected their dexterity and surgical skills [1]. Both speed and quality of performance were measured as outcomes [1]. The study concluded that musicality could be a marker for basic surgical skills development in identifying potential surgical trainees [1]. Another study by Moglia et al. which was also conducted on medical students concluded that the psychomotor skills of participants that were exposed to both musical instrument playing and video games were higher than those that were only exposed to either one alone for performances at a simulator for robotic surgery [4]. Studies by Moustaki et al., Hazboun et al., and Harrington et al. included musical instrument playing as one of the predictors of performance in minimally invasive surgery [5, 6, 7]. The study by Margarita et al. found a significant difference in the scores obtained in completing a microsurgical anastomosis between novice trainees that played musical instruments compared to those that did not play, while the study by Hazboun et al. found that pediatric surgery applicants with musical instrument playing background demonstrated significantly faster knot times compared to those without prior exposure [5, 6]. The study conducted by Heather et al., also conducted on medical students found that those with musical instrument playing exposure had a significant positive association with skills acquisition when asked to perform several surgical tasks [3, 7].

And yet, some other studies found no association between Musical instrument playing and surgical skills acquisition utilized laparoscopic surgical skills as the measured outcome, where those with prior exposure did not outperform [8, 9, 10]. A study by Osborn et al. conducted on medical students and undergraduates that assessed their surgical skills by performing a microsurgical task using a novel simulator found that amongst musicians, those who started playing at a younger age obtained higher scores than those who began playing later [11]. However, the study concluded that those with musical instrument playing exposure did not obtain higher scores than those without exposure [11]. While many of these studies did not involve studying surgical skills acquisition in the long‐term, a prospective study by Yamacake et al. analyzed the learning curve for transurethral resection of the prostate (TURP) amongst second year Urology students with a prior musical instrument or video game exposure [12]. This 4‐year study concluded that musical instrument playing did not affect TURP performance [12].

Currently, there is insufficient evidence to describe the association between musical instrument playing and surgical skills acquisition. This is reflected by the small sample sizes of existing studies and the lack of characterization of participants' musicality through the type of instrument played, years of exposure, and frequency of playing. Existing studies are also limited by the selection bias resulting from recruiting participants that self‐volunteered and the high attrition rates. Furthermore, besides the limited range of surgical skills tested, existing studies also lack validated and standardized scaling measurements in measuring surgical skills. Although many studies required participants to learn new surgical techniques, none of the studies have followed up with their participants to assess their skills retention.

In view of the conflicting results of previous studies, the current study aims to evaluate the association between basic surgical skills and medical students' experience with playing musical instruments.

## 2 Methods

**FIGURE 1:**
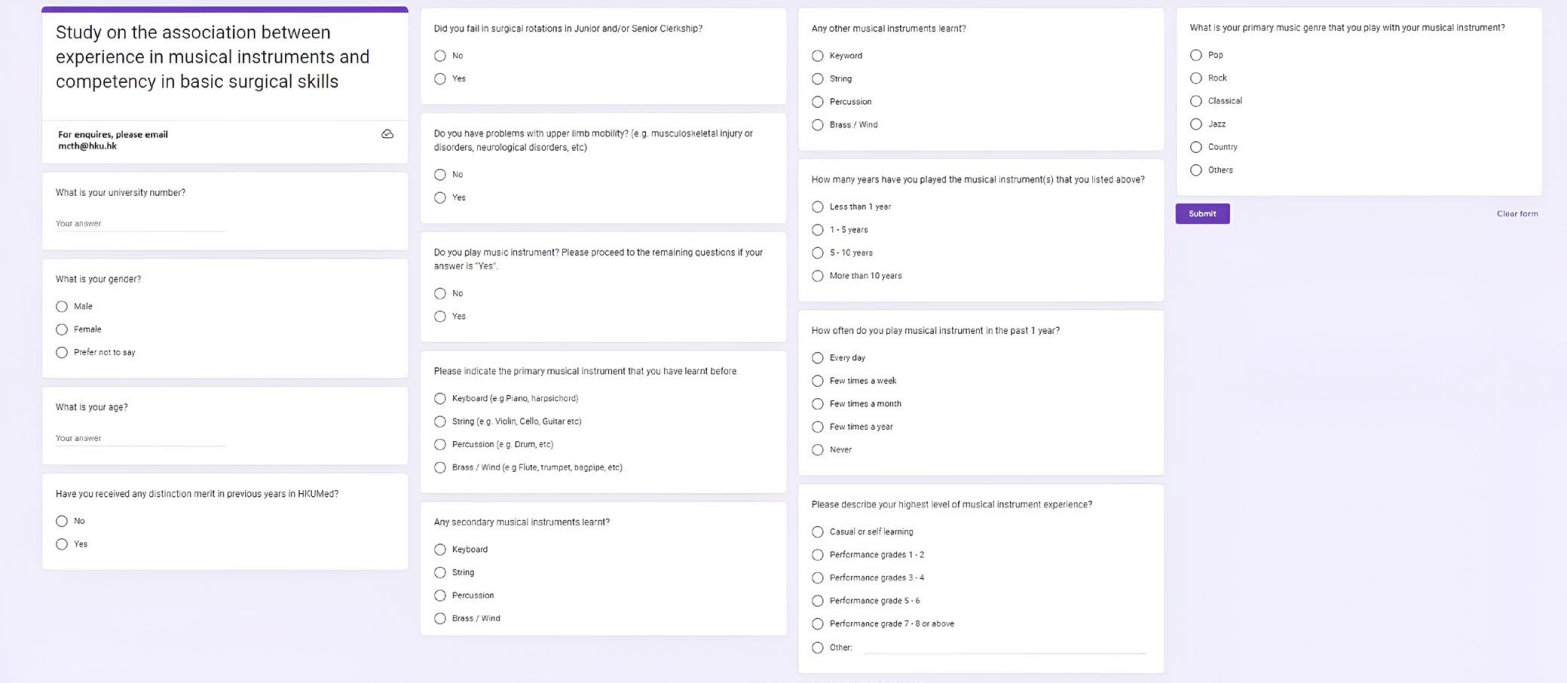
Online anonymous questionnaire for data collection from medical students.

This is a cross‐sectional study with institutional approval for data collection. Consents were obtained from students. Participation was voluntary. Two hundred three final year medical students were recruited into the study. An online questionnaire was distributed to the students on their experience in musical instruments. Additional relevant information such as types of instruments played, years of experience, level of experience, and their current activity in playing musical instruments were also asked. Please refer to Figure 1 for details.

Basic surgical skills teaching sessions were delivered in small groups of 30–35 students, in the form of a two‐hour interactive tutorial. All sessions were taught by a single surgical teacher (MC) to ensure consistency of the contents delivered. Topics covered include (A) surgical instruments handling (scalpel, scissors, needle holder, surgical forceps), (B) creating a linear incision for lumps and bumps excision, (C) suturing of wounds with instrumental knots, (D) creating surgical knots by hand‐tie, and (E) cutting of threads.

Surgical instruments and synthetic skins were distributed to students to practice at home prior to the Objective structured clinical assessment (OSCA) of their basic surgical skills 4 weeks after the teaching session.

**FIGURE 2:**
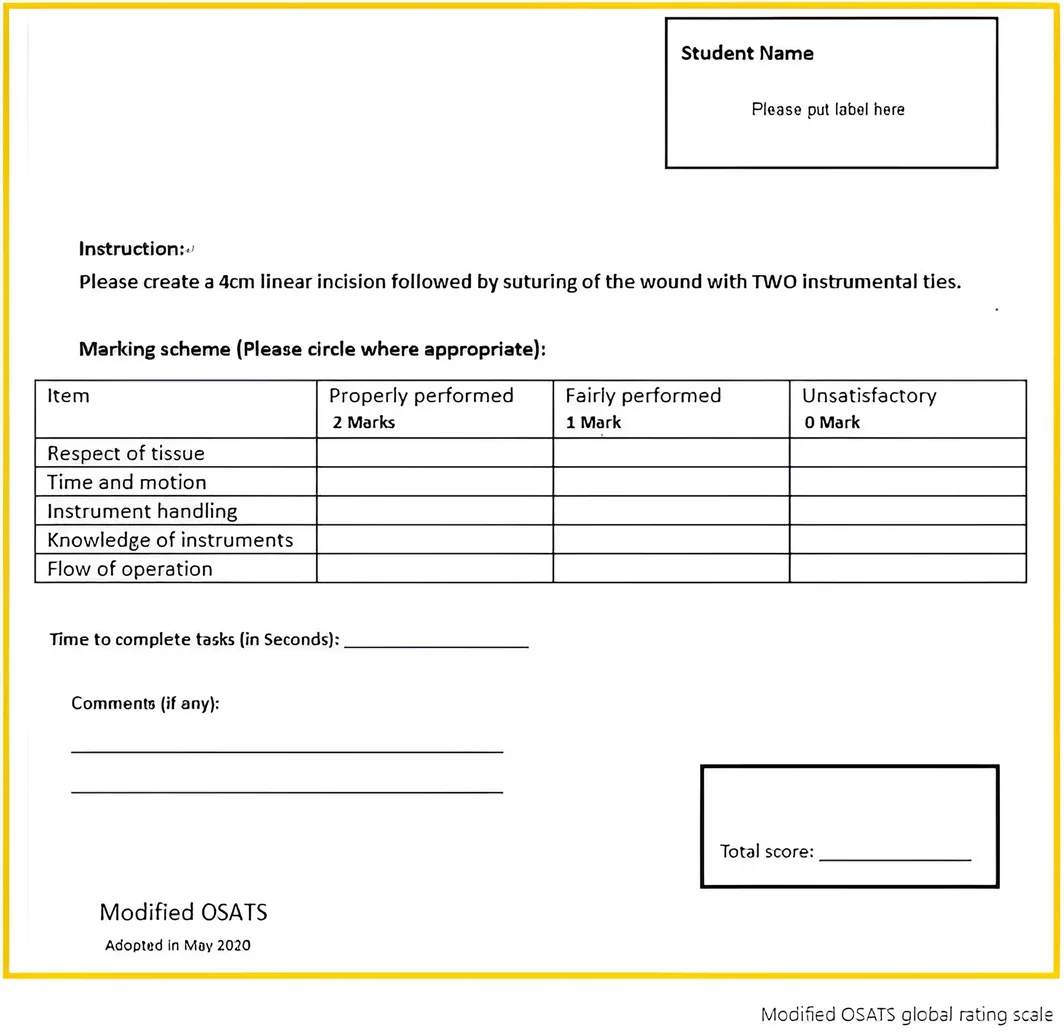
Marking scheme for objective assessment of medical students' surgical skills.

OSCA on basic surgical skills was conducted by two blinded assessors (MC and BC) who are both qualified specialists in General Surgery; assessors were blinded to students' experience in musical instruments and other demographic factors. Students were instructed to create a 4 cm linear incision followed by wound suturing with instrumental knots. The surgical skills of the students were assessed by Objective Assessment of Technical Skills (OSATS) (Figure 2) [13]. Time to complete the tasks was recorded (from incision to thread cutting, in seconds).

Students' OSCA scores and their respective experience in musical instruments were stored into a prospective database by a blinded administrative assistant. The database was then analyzed. Students who had experience in playing musical instruments were grouped into the case group, where those who do not have any experience in playing musical instruments were grouped into the control group. Surgical skills between the two groups were then compared.

## 3 Results

### 3.1 Baseline Demographic Data

Two hundred three final year medical students were recruited into the study. Ninety nine were female students and 104 were male students. The median age was 24 years old (range 23–34). Seventeen (8.4%) students had a history of obtaining merits/distinction awards in Surgery examinations in previous years. While 9 (4.4%) students had a history of failing surgical examinations.

Seventy seven (37.9%) students had no experience with playing any musical instruments, while 126 (62.1%) had experience with playing musical instruments. Of which, 95 (75.4%) mainly played classical music, while 31 (24.6%) mainly played pop music.

### 3.2 Information on Musical Instruments Played

One hundred one (80.2%) students played keyboard instruments (such as piano) as their primary instrument. Twenty three (18.3%) played strings (such as violin or cello) as their primary instrument. Two (1.5%) played brass/wind instruments as their primary instrument. None of the students played percussion (such as drums) as their primary musical instruments. Sixty eight (54.0%) students indicated that they have learnt more than one type of musical instrument.

Concerning the level of experience with musical instruments. One hundred six (84.1%) students had achieved Grade 7 or above in professional music examinations. Nineteen (15.1%) had achieved Grade 6 or below in professional musical examinations. One (0.8%) student reported to have acquired skills in playing guitar through self‐learning/casual practice.

Fifty six (44.4%) students reported to have frequently practiced their musical instruments (defined as playing at least once a week). Fifty one (40.5%) students reported to have infrequent practice of musical instruments (defined as playing less than once a week). Nineteen (15.1%) students reported to have never played or practiced a musical instrument in the past 12 months.

### 3.3 Surgical Competency and Experience in Musical Instrument Playing

The mean time for task completion was 249.3 s (SD = 45.9) in the control group (no musical experience), while that of the case group (with musical experience) was 223.5 s (SD = 28.8), *p* < 0.001. Hence, students with musical training completed the task 25.9 s faster (95% confidence interval [CI]: 14.3–37.4 s) with a standardized effect size around 0.7 SD (CI 0.42–1.00).

A multiple linear regression was calculated to predict time for task completion based on gender, age, academic distinction, previous surgical failure, and musical instrument playing. Only musical instrument playing and prior distinction were statistically significant. Musical instrument playing was the strongest predictor with a mean difference of −26.7 s (CI: −37.1, −16.4 s, *p* < 0.001). This indicated that musical instrument players completed tasks significantly faster than non‐musicians (Cohen's *d* = −0.75, CI: –1.04, −0.46), signifying a medium–large effect. On the other hand, prior distinction was unexpectedly associated with longer task completion time with a mean difference of 24.6 s (CI: 5.2–43.9 s, *p* = 0.013). This corresponds to a standardized effect of Cohen's *d* = 0.69 (CI: 0.15–1.23), signifying a medium effect.

The mean OSATS score was also compared. In the control group, the score was 7.79 (SD = 0.78) while that in the case group was 8.28 (SD = 0.59), *p* < 0.001. The control group had 0.49 marks (CI: 0.28–0.69) lower than the case group, with an effect size of 0.73 (CI: 0.43–1.02).

A multiple linear regression was conducted comparing the other factors listed above in relation to the OSATS score. Playing musical instruments remained the only statistically significant factor (*p* < 0.001) correlated with the OSATS score after adjusting for confounders, with a mean difference of 0.49 (CI: 0.30–0.69). The effect size was moderate to large with a Cohen's d of 0.74 (CI: 0.45, 1.02). None of the other predictors (gender, age, distinction, or surgical failure) was significantly associated with OATS scores (all *p* > 0.05).

### 3.4 Surgical Competency and Types of Musical Instruments Played

Majority of medical students with exposure to musical instrument training played keyboard or strings as their primary instrument. A comparison was made between these two.

The mean time for task completion was 223.8 s (SD = 30.8) in the keyboard group, while that in the string group was 223.2 s (SD = 18.6), *p* = 0.926. The mean OSATS score was 8.3 (SD = 0.59) in the keyboard group and 8.26 in the string group (SD = 0.54), *p* = 0.789.

Fifty eight students played only one musical instrument, while 68 students had experience in playing more than one musical instrument. The mean time for task completion was 223.4 s (SD = 29.6) and 223.5 s (SD = 28.4) respectively, *p* = 0.99. The mean OSATS scores were 8.31 (SD = 0.6) and 8.25 (SD = 0.6) respectively, *p* = 0.57.

### 3.5 Surgical Competency and Grades in Professional Qualifications in Musical Instrument Playing

One hundred six (84.1%) students had achieved Grade 7 or above in professional music examinations. The median OSATS score was 8.15 (SD = 0.59), while those who achieved Grade 6 or below in professional music qualifications had a median OSATS score of 8.30 (SD = 0.59), *p* = 0.291. Likewise, the median time for task completion of the 73 students who achieved Grade 7 or above was 223.0 s (SD = 28.8), while those who achieved Grade 6 or below had a median time of task completion of 226.1 s (SD = 29.8), *p* = 0.664.

### 3.6 Comparing Surgical Competency Between Active Players and Non‐Active Players

Fifty‐six (44.4%) students reported having frequent playing of musical instruments (defined as playing musical instruments for at least once per week). The mean time for task completion was 220.3 s (SD = 23.4) and the median OSATS score was 8.4 (SD = 0.6). 70 (55.6%) students reported having infrequent playing of musical instruments (defined as playing musical instruments less than once per week) or even never played for the past 12 months; the median time for task completion was 226.0 s (SD = 32.5), *p* = 0.275, and the median OSATS score was 8.2 (SD = 0.6), *p* = 0.177.

## 4 Discussion

The concept of cross‐domain transferability of skills has gained increased recognition over the years [14]. Notably, the association between the abilities acquired through musical training and that for surgical skills has been a focal point of several studies. The parallels drawn between the two disciplines revolve around the shared need for precision, hand‐eye coordination, dexterity, and the ability to perform under pressure [3, 15]. In the context of this evolving literature, our study aimed to analyze the surgical performance of medical students with varying levels of musical training and found intriguing results that contribute to the ongoing discourse.

A study by Graziano et al. suggested that surgical novices with a background in music demonstrated superior performance in fundamental surgical skills, particularly regarding technical quality [3]. This resonates with our study, where we observed an enhanced ability to execute surgical tasks quicker among students with a musical background. In addition, a significant difference in surgical skills between students with or without prior musical background was also demonstrated in our study. This significant difference may be attributed to the transferability of skills required in playing musical instruments and in acquiring basic surgical skills. The findings of this study reflect that students who had prior musical background may acquire basic surgical skills faster than their counterparts who did not have prior exposure. This may be considered for in surgical residents' recruitment, where residents with prior musical background may acquire basic surgical skills faster and move on to learning more complex surgical skills earlier than their colleagues without prior surgical background.

However, the relationship between musical training and surgical performance may not extend uniformly to more complex procedures. A recent literature review analyzed the influence of musical experience on laparoscopic surgical skill performance and showed no difference in the performance outcomes [16]. Laparoscopic surgery is considered more advanced and complex due to the fulcrum effect (whereby instrument movements are reversed at the trocar insertion point), two‐dimensional visualization of a three‐dimensional surgical field, and reduced haptic feedback [17]. All of which create unique perceptual and motor demands. Hence, the performance on complex surgery may rely on different skill sets rather than those enhanced by musical training. Likewise, the mismatch of findings in Kleiton et al.'s study on TURP learning curve and our study's finding may reflect task specificity. The skills acquired in musical training may be transferable to isolated dexterity‐based tasks, such as the simple surgical tasks used in our study [3], whereas the skills required in TURP may require more visuospatial and procedural learning to acquire [12]. Future longitudinal studies should examine whether musicians maintain their advantage when progressing to advanced surgical techniques, while carefully controlling for potential confounders such as deliberate practice hours outside of formal training.

Among musicians, our study did not find any significant association between the level of musical instrument experience, practice frequency, and the speed or quality of surgical task performance. This suggests that while learning to play a musical instrument and acquiring basic surgical skills may rely on similar underlying abilities, additional musical practice does not necessarily translate into improved surgical performance. Reflecting that perfecting skills in musical instrument playing and surgical skills will require more specific skills‐based practice.

Our research did not find a significant difference in task performance between different subgroups of musical instruments, namely instruments played from the family of keyboard and string instruments. This resonates with the findings of Graziano et al., where the type of instrument, among other factors, did not correlate with differences in surgical task performance [3]. This suggests that the benefits derived from musical training might not be instrument‐specific but rather tied to the generic skills acquired from such training.

Interestingly, our results showed that students who had received distinctions took significantly longer at 24.6 s (CI: 5.2–43.9 s, *p* = 0.013) to complete the surgical tasks. While some might expect high‐achieving students would be able to complete the task faster, a growing body of literature suggests that the relationship between skill and task completion time is complex and context‐dependent [18]. Goldhammer et al. demonstrated that time‐on‐task effects are moderated by both task difficulty and individual skill level, with more skilled individuals sometimes requiring more time on tasks requiring controlled cognitive processing [18]. In problem‐solving contexts requiring deliberate analytical thinking, spending more time may reflect greater thoroughness and careful consideration rather than inefficiency. This interpretation is supported by research showing that high‐performing students sometimes exhibit longer execution times on complex tasks, as they engage in more exhaustive cognitive processing [19].

While our study has provided insightful results, it is not without limitations. Firstly, our cohort was restricted to students from a single institution and class, potentially affecting the external validity of our findings. Secondly, despite achieving an impressive response rate with a decent sample size of 203 subjects, it is still relatively difficult to conduct a subgroup analysis on some types of instruments (such as Brass/Wind and Percussion), which are not commonly played in our cohort of students. Another limiting factor would be the inability of the questionnaire to assess how much practice students had outside of the classroom and previous exposure in surgical skills training in the earlier years of medical school, which are both potential confounding factors. Furthermore, while our research utilized a comprehensive questionnaire to assess participants' musical backgrounds, responses may still be subject to recall bias. Additionally, as a cross‐sectional study using a single exam as an endpoint, our ability to assess our participants' full range of surgical skills was constrained. The design of this study prevents causal inference and does not allow for assessment of longitudinal learning curves or retention. For future studies, it would be beneficial to include additional outcome measures. While our study found that students with musical backgrounds had enhanced ability to execute surgical tasks quicker, this finding may not necessarily relate to clinical patient outcomes. Evaluating students' performance in a broader range of tasks and, more importantly, linking their surgical skills to patient outcomes could provide a more comprehensive understanding of the potential impact of musical training on surgical proficiency. In addition, expanding this study to include other activities that require dexterity such as video games and sports may provide new insights to this topic of research. Adopting a longitudinal study design may provide more insight into how training in musical instruments impacts surgical skills. Nevertheless, our results and conclusions were drawn from a carefully designed cross‐sectional study with standardized assessment of students' basic surgical skills by independent blinded assessors, findings from this study add additional evidence to support the positive association between experience in playing musical instruments and surgical skill acquisition.

## 5 Conclusion

Medical students with prior exposure to musical instruments training were able to complete basic surgical tasks quicker and with better technique when compared to their peers without experience in playing musical instruments.

## Funding

The authors have nothing to report.

## Conflicts of Interest

The authors declare no conflicts of interest.

## Data Availability

The data that support the findings of this study are available from the corresponding author upon reasonable request.
